# Impact of Endocrine Disorders on IVF Outcomes: Results from a Large, Single-Centre, Prospective Study

**DOI:** 10.1007/s43032-022-01137-0

**Published:** 2022-12-07

**Authors:** Tunde Herman, Szilvia Csehely, Monika Orosz, Harjit Pal Bhattoa, Tamas Deli, Peter Torok, Antonio Simone Lagana, Vito Chiantera, Atilla Jakab

**Affiliations:** 1 Assisted Reproduction Centre, Clinical Centre, University of Debrecen, Egyetem Tér 1, 4032 Debrecen, Hungary; 2 Department of Obstetrics and Gynaecology, Faculty of Medicine, University of Debrecen, Egyetem Tér 1, 4032 Debrecen, Hungary; 3 Department of Laboratory Medicine, Faculty of Medicine, University of Debrecen, Egyetem Tér 1, 4032 Debrecen, Hungary; 4 Department of Health Promotion, Mother and Child Care, Internal Medicine and Medical Specialties (PROMISE), University of Palermo, Piazza Marina, 61, 90133 Palermo, Italy

**Keywords:** Assisted reproduction, In vitro fertilization, Endocrine diseases, Thyroid diseases

## Abstract

Endocrine disorders negatively influence the ovarian function, and increasing incidence of endocrine diseases with age may have further negative effects on pregnancy rate. Prospective cohort study of 231 consecutively enrolled patients underwent IVF treatment. In patients with known endocrine disorders, the laboratory parameters were corrected before IVF treatment. One hundred sixty one patients (69.7%) had at least one known and treated endocrine disorder (study group), and 70 patients were endocrine negative (control group). Endocrine disorders diagnosed were thyroid disorders (32.5%), diminished ovarian reserve (23.8%), insulin resistance (22.5%), PCOS (15.2%), hyperprolactinaemia (13.4%), obesity (12.1%), hypogonadotropic hypogonadism (0.8%) and congenital adrenal hyperplasia (0.2%). Before the IVF treatment, systematic endocrine laboratory examinations were performed in all patients. Higher age, BMI and FSH were found in the study group, while AMH level was lower. There were no differences in LH, E2, prolactin, TSH, FT3, FT4, TT, DHEAS, androstendione, 17-OHP and SHBG level between the study and control groups. The study group had higher baseline glucose, baseline insulin, 120-min glucose and 120-min insulin level after oral glucose tolerance test. With no difference in the IVF cycles performed, pregnancy rate was lower in the study group (61.43% vs. 34.16%; *p* = 0.003), and this difference (*p* = 0.0151) remained in age-corrected rates, as well. The analyses were also performed in individual endocrinology groups. The prevalence of endocrine disorders is high in females participating in IVF programs, and they are often accompanying each other. Even after proper correction, the presence of the endocrine disorder negatively influences the pregnancy rate in IVF treatment.

## Introduction

The rate of infertility is increasing worldwide. Healthcare providers must manage difficulties in reproduction caused by endocrinological changes or disorders, delayed childbearing, negative environmental effects, as well as changes in lifestyle and nutrition. The prevalence of infertility has significantly increased over the past decades, affecting 8–12% of couples of reproductive ages [1].

The primary indications for in vitro fertilization (IVF) were anatomical disorders of the female reproductive tract (occlusion of the fallopian tubes and endometriosis). Poor semen quality is the sole cause of infertility in 20% of couples and contributes to fertility issues in another 20% [2]. With the development of the intracytoplasmic sperm injection effective treatment for male infertility has become widely available. Diminished ovarian reserve (DOR) and increased genetic damage of the oocyte pool are also significant indications for in vitro fertilization (IVF) resulting from delayed childbearing. Thus, the order and the distribution of the main indications for IVF are as follows: male factor infertility (35%), DOR (30%), fallopian tube obstruction (14%), ovulatory dysfunction (14%), endometriosis (9%) and uterine factor (5%). Male and female factors are simultaneously present in 18% with 11% associated female factors. Other known causes are present in 13%, while in 12% of the cases the cause of infertility is unknown. As for the females, DOR and chronic anovulation can be the most frequent indications for IVF [3].

Increasing incidence of endocrine diseases with age may have further negative effects on fertilization rate. In the case of ovulatory dysfunction, ovulation induction treatment usually restores fertility. Among the endocrine disorders causing ovulatory dysfunction in patients enrolled to assisted reproduction programs, marked prevalence of thyroid disease, polycystic ovarian syndrome (PCOS), diminished ovarian reserve (DOR) and hyperprolactinaemia have been observed [4].

Among women of reproductive age, hypothyroidism is a common cause of infertility due to anovulation, with a prevalence of 3–5% [5]. Worldwide, the prevalence of Hashimoto’s thyroiditis, which increases by age, is currently between 8 and 14% among women of reproductive age [6], and the incidence of autoimmune thyroid disease (AITD) is even higher among infertile women [7]. According to the patients’ registers at the centres of assisted reproduction, the incidence of thyroid autoimmunity (AITD) may be as high as 20% [8].

DOR is the only endocrine disorder that is an absolute indication of IVF. The incidence of DOR increases by age, and its prevalence among patients requesting IVF in the USA grew from 19 to 26% in the period of 2004–2011 [9].

PCOS, the most common endocrinopathy and a main cause of infertility among women of reproductive age, has a prevalence of 5–20%, due to varying ethnicities and diagnostic criteria. PCOS is a complex clinical condition, affecting both reproduction and the metabolic processes [10–12]. Although ovulation can usually be restored by sorting out etiological causes and providing treatment to induce ovulation, success rates from assisted reproductive technologies (ART) may be lower because of the presence of underlying endocrine and metabolic disorders.

In addition to hyperprolactinaemia associated with PCOS, the prevalence of metabolic hyperprolactinaemia is also on the rise, similar to other forms of the disease such as drug-induced and liver-function-related hyperprolactinaemia — due to stressful lifestyles, as well as eating disorders [13].

Except for DOR, endocrine disorders are not the leading indications for ART, but their treatment prior to ART is necessary to achieve optimal results. However, there is little data available to estimate to what extent ART can be successful in association with endocrinopathy. The primary aim of this study is to investigate how the presence of treated endocrine disorders affects pregnancy rates in the IVF practice, irrespective of the patients’ age, whereas the secondary aim is to examine the prevalence of the different endocrine diseases among IVF patients and what is their specific impact in IVF programs.

## Patients and Methods

We conducted a prospective analysis of 231 consecutively enrolled women (mean age: 34 years, range: 21–44 years) who underwent IVF treatment at our centre. A thorough endocrinological investigation of each patient requesting IVF treatment in this cohort was performed. The patients were considered overweight with a body mass index (BMI) over 25, and obese in case of BMI of more than 30. Serum cholesterol and triglyceride were recorded. As part of the infertility work-up, regardless of IVF indication, we tested the following endocrinological parameters: serum levels of follicle stimulating hormone (FSH), luteinizing hormone (LH), estradiol (E2), prolactin (PRL) supplemented with macroPRL if PRL was elevated, thyroid stimulating hormone (TSH), fT3, fT4, thyroid peroxidase antibody (TPOAb), thyroglobulin antibody (TGAb), total testosterone, fasting glucose and insulin, 21-OH vitamin D and anti-Mullerian hormone (AMH) on days 2–4 of the menstrual cycle. In case of elevated testosterone levels or clinical signs of hyperandrogenism, we also assessed other androgen hormone levels (dehydroepiandrosterone sulfate: DHEAS; androstenedione and 17-hydroxi-progesterone) and sex hormone binding globulin (SHBG). Following menstruation, as part of a detailed transvaginal ultrasound examination, antral follicular count (AFC) measurements and evaluation of the ovaries’ morphology were performed to diagnose DOR or establish polycystic ovarian morphology (PCOM). In the absence of regular periods or when amenorrhea was present, the evaluations were carried out independent of the day of the cycle. Any previous endocrine therapy was also documented in the patients’ records.

If we suspected abnormal thyroid function from laboratory tests, a thyroid ultrasound was performed, and if TSH levels were low (< 0.35 mU/l), TSH receptor antibodies (TRab) was also measured.

We classified (subclinical) hypothyroidism, Hashimoto’s thyroiditis, hyperthyroidism and nodular or malignant lesion as thyroid diseases. The diagnosis of thyroid autoimmunity was based on elevated levels of TPOAb or TGAb, according to the local laboratory reference values (TPOAb > 16 U/ml and TGAb > 60 U/ml). Thyroid autoimmunity accompanied by euthyroidism and normal thyroid ultrasound scan was not included among thyroid diseases, but there was a precondition: before and during IVF treatment, the target value of TSH had to be below 2.5 mU/l, and the treatment was modified accordingly. Hypothyroidism and subclinical hypothyroidism were established according to the current clinical guidelines (TSH cut-off > 4.0 mIU/l), when fT4 was normal and based on two measurements [14, 15]. Hashimoto’s thyroiditis was diagnosed when thyroid autoimmunity was accompanied by (subclinical) hypothyroidism, or the ultrasound scan confirmed the disease.

The diagnosis of PCOS was established according to the Rotterdam criteria [16], with consideration to new additions by different associations [17, 18]. We also followed the Rotterdam criteria when providing the sonographic description of PCOM.

The Bologna criteria served as a basis to define the levels of hormones (FSH, LH and E2) for diagnosing diminished ovarian reserve (DOR), together with AMH and AFC (Ferraretti et al., 2011). Based on a sonogram, low AFC was diagnosed if less than four antral follicles per ovary were imaged considering the age-adjusted nomogram (Almog et al., 2011).

Hyperprolactinaemia was defined as elevated PRL after having taken away the macroPRL. If PRL exceeded 100 ng/ml, magnetic resonance imaging (MRI) of the sella was performed.

Oral glucose tolerance test (OGTT; 0, 60 and 120-min measurements of glucose and insulin) was performed to reveal insulin resistance (IR). As no diagnostic consensus is currently available, we followed the recommendation suggesting that a patient has IR or severe IR if the 60-min value is over 80 or over 300, respectively, or the 120-min value is over 55 or over 100, respectively. Impaired glucose tolerance (IGT) and diabetes mellitus (DM) were identified according to textbook recommendations (Taylor et al., 2020).

MRI evaluation of the sella and an investigation of the pituitary gland’s hormone secretion (FSH, LH, TSH, ACTH, GH and PRL) served as the basis of making the differential diagnosis of central hypogonadotropic hypogonadism.

Hyperandrogenic symptoms and elevated 17-hydroxyprogesterone (17-OHP) levels prompted us to carry out an adrenocorticotropic hormone (ACTH) stimulation test for verification of congenital adrenal hyperplasia (CAH).

First, we investigated the incidence of various endocrinological abnormalities in women participating in the IVF program and their distribution according to the primary IVF indications. IVF treatments were performed after the necessary endocrinological therapeutic correction was completed. The study group consisted of patients with endocrine disorders. Those without endocrinological abnormalities were considered as the control group. We compared the patients in both the study and control groups as well as the subjects in the study group with different endocrine disorders by age, BMI, laboratory parameters (AMH, FSH, LH, E2, prolactin, sTSH, FT3, FT4, total testosterone, DHEAS, androstenedione, 17-OHP, SHBG, glucose and insulin at 0, 60 and 120 min), the number of oocytes retrieved (NOR), clinical pregnancy rate and the number of cycles required to achieve pregnancy. We also evaluated the outcomes of IVF treatments in each group after age adjustment.

During IVF treatment, patients were monitored and managed according to our standardized clinical protocol. The stimulation protocol and recombinant follicle-stimulating hormone dose were determined on an individual basis according to characteristics of the patient’s basic hormone levels and AMH level. Patients underwent transvaginal ultrasonography and hormonal monitoring during hyperstimulation three times. When the leading follicle reached 18 mm, 250 μg of recombinant human chorionic gonadotropin (hCG) was administered subcutaneously. Oocyte retrieval was performed transvaginally 36 h after hCG injection. Embryo transfer was performed with 1 or 2 embryos on day 3 or 5. Patients received vaginal, oral and subcutaneous progesterone supplementation therapy along with low-molecular-weight heparin (LMWH) therapy. On day 14, serum hCG levels were measured. Pregnancy was defined when hCG level was above 50 mIU/ml.

The research was approved by the Regional and Institutional Research Ethics Committee of the Clinical Centre of the University of Debrecen (DE RKEB/IKEB 5684–2021). The design, analysis, data interpretation, drafting and revisions followed the Helsinki Declaration and strengthened the reporting of observational studies in epidemiology (STROBE) statement, available through the enhancement of the quality and transparency of health research (EQUATOR) network (www.equator-network.org). Each patient enrolled in this study signed an informed consent form for all procedures and to allow data and biological sample collection and analysis for research purposes. No remuneration was offered for study participation.

### Statistical Analysis

For descriptive statistics, we used absolute and relative frequencies for categorical variables and mean and standard deviation for continuous variables. Associations between categorical outcomes and other variables were assessed using Fisher’s exact tests in unadjusted analysis and logistic regression in analysis adjusted for confounders. Patient groups were compared in terms of continuous outcomes using Student’s *t* tests (if distributional assumptions were satisfied) or Wilcoxon’s rank sum tests (otherwise). The significance criterion was set at the conventional *p* < 0.05. Data handling and analysis were performed using version 15 of the Stata statistical package (StataCorp. 2017. Stata Statistical Software: Release 15. College Station, TX: StataCorp LLC).

## Results

### Main Indications of IVF Treatment

Of the 231 patients enrolled in this study, the main indications for IVF treatment in order of frequency were as follows: male factor (*n* = 70; 30.3%), DOR (*n* = 55; 23.8%), tubal factor (*n* = 43; 18.6%), chronic anovulation (*n* = 32; 13.8%), unexplained idiopathic infertility (*n* = 18; 7.7%) and endometriosis (*n* = 13; 5.6%).

### Endocrinological Disorders as Main or Co-indications for IVF

Endocrinological diseases were identified in 161 patients (69.7%, average age: 34.7 years, range: 19–45), while 70 patients (30.3%, average age: 32.3 years, range 21–44) had no underlying endocrine disorder. The main indication for IVF treatment was anovulatory dysfunction in 87 cases (DOR *n* = 55, PCOS *n* = 29, hypogonadotropic hypogonadism *n* = 2 and congenital adrenal hyperplasia *n* = 1), while in 74 cases endocrinological disease appeared as a co-factor.

### Prevalence and Association of Endocrinological Disorders in IVF Patients

The breakdown of underlying endocrinological disorders among IVF patients was as follows: thyroid disorders (*n* = 75/231, 32.5%), DOR (*n* = 55/213, 23.8%), IR (*n* = 52/231, 22.5%), PCOS (*n* = 35/231, 15.2%), hyperprolactinaemia (*n* = 31/231, 13.4%), obesity (*n* = 28/231, 12.1%), hypogonadotropic hypogonadism (*n* = 2/231, 0.8%) and congenital adrenal hyperplasia (*n* = 1/231, 0.2%).

Thyroid disorders represented almost half (46.6%) of the endocrine-positive patients. Among patients with thyroid disease, the most frequent disorder was Hashimoto’s thyroiditis (52 cases, 68%). There were 15 (20.0%) cases of hypothyroidism without laboratory or ultrasound evidence of autoimmune thyroid disease (AITD). Graves’ disease was found in 3 cases, acute thyroiditis in 1 and thyroid cancer in 1 case.

DOR was diagnosed in 55 women, which accounted for 23.8% of all patients enrolled in the study and for 34.16% of patients with endocrinopathy. Concomitant endocrine comorbidity was found in 39 cases (70.9%) of patients with DOR assessed for polyglandular endocrinopathy. Most frequent disorders were thyroid disease (*n* = 21, 38.18%), Hashimoto’s thyroiditis (*n* = 14, 25.45%), IR (*n* = 10, 18.18%) and hyperprolactinaemia (*n* = 6, 10.9%). Only 29.09% of the DOR patients had no co-existing endocrine disorder.

PCOS was detected in 15.2% (*n* = 35) of patients overall, which accounted for 21.7% of the endocrine-positive group. In 14 cases (40%) thyroid disease was diagnosed as a co-morbidity for PCOS, of which Hashimoto’s thyroiditis was present in 7 cases (20%). IR was detected in 68.6%, overweight (BMI over 25) in 54% and obesity (BMI over 30) in 17.1%.

Hyperprolactinaemia was diagnosed in 31 patients (13.4% of all patients and 19.3% of endocrine-positive patients), in whom hypophyseal microadenoma was diagnosed in 4 cases (12.9%) with MRI. PCOS and IR co-morbidities were both present in 6 cases (19.4%). In 19.4% of the cases with hyperprolactinaemia, drug use (anti-epileptic therapy: 1; antidepressant therapy: 2 and antihypertensive therapy: 3) was the underlying reason. There was no specific reason of hyperprolactinaemia identified in 48.4% of the cases. Thyroid disease in 45.16%, Hashimoto’s thyroiditis in 15.38% and DOR in 19.35% were found as comorbidities in this group.

### Comparison of Clinical and Hormonal Parameters and IVF Outcomes

Clinical and laboratory features of the study group and subgroups and the controls are summarized in Table 1.

**Table 1:**
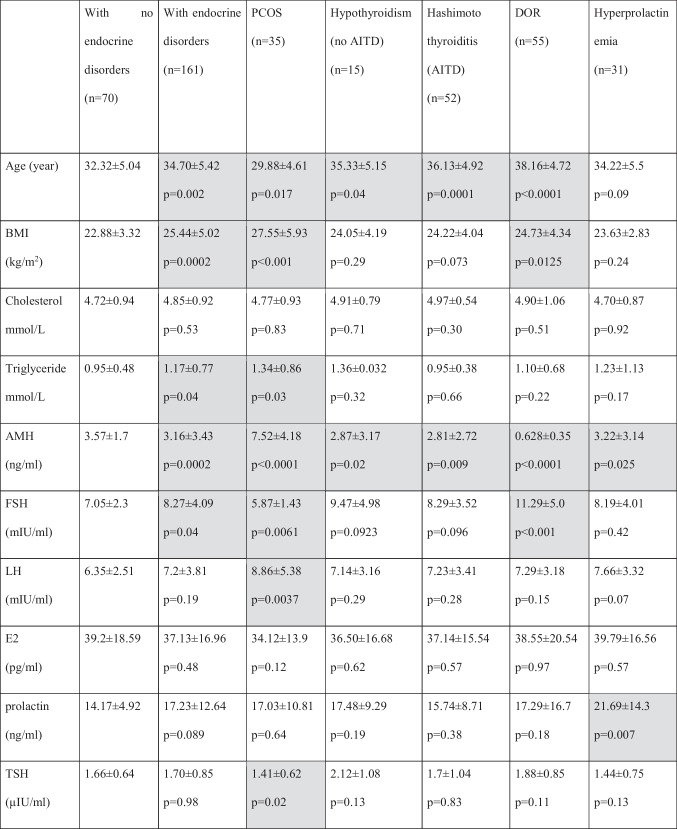

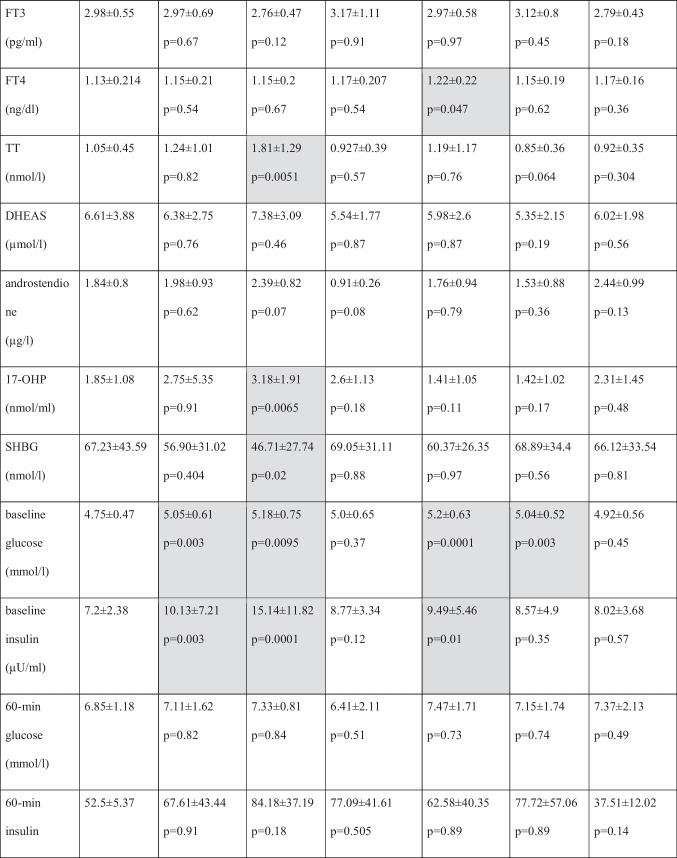

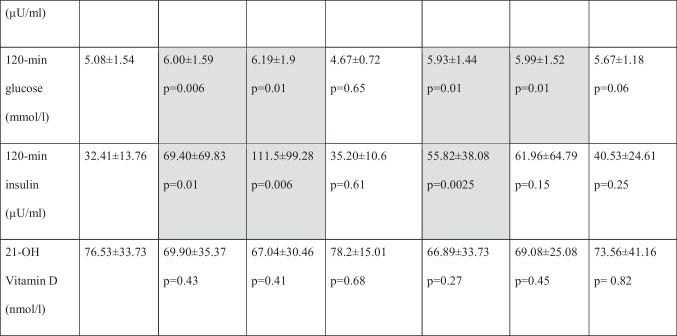
Clinical and laboratory features of studied groups

Values represent arithmetic means. *P* values pertain to comparisons between patients defined in column headers and patients with no endocrine disorders

*17-OHP* 17-hydroxyprogesterone, *AITD* autoimmune thyroiditis, *AMH* anti-Müllerian hormone, *BMI* body mass index, *DHEAS* dehydroepiandrosterone sulfate, *DOR* diminished ovarian reserve, *E2* estradiol, *FSH* follicle stimulating hormone, *FT3* free triiodothyronine, *FT4* free thyroxine, *LH* luteinizing hormone, *min* minute, *PCOS* polycystic ovarian syndrome, *SHBG* sex hormone binding globulin, *TSH* thyroid stimulating hormone, *TT* testosterone

IFV outcomes of the study group and subgroups and the controls are summarized in Table 2.

**Table 2:**
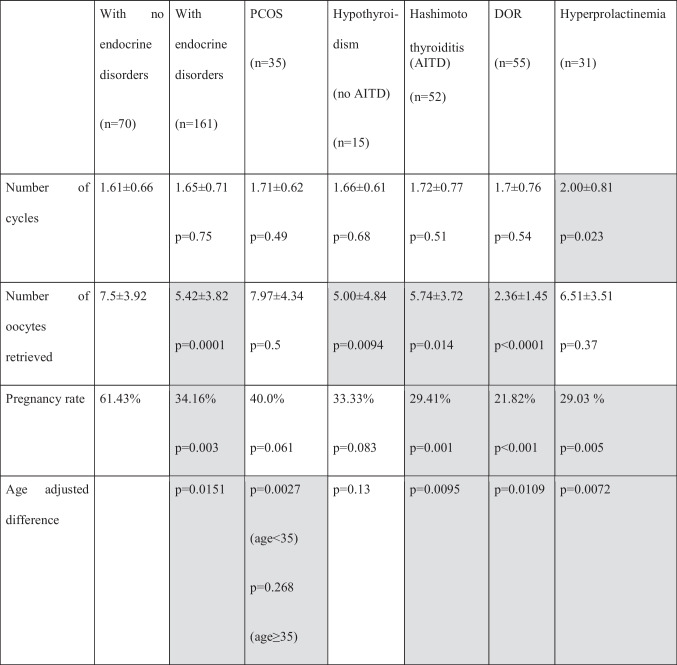
IVF outcomes of studied groups

Values represent arithmetic means or percentages. *P* values pertain to comparisons between patients defined in column headers and patients with no endocrine disorders

*AITD* autoimmune thyroiditis, *DOR* diminished ovarian reserve, *PCOS* polycystic ovarian syndrome

### Comparison of Infertile Women with and Without Endocrinopathies

Average age (32.3 ± 5.04 vs. 34.7 ± 5.42 years; *p* = 0.002), BMI (22.88 ± 3.32 vs. 25.44 ± 5.02 kg/m^2^; *p* = 0.0002) and serum triglyceride level (0.95 ± 0.48 vs. 1.17 ± 0.77, *p* = 0.04) were significantly higher in endocrine-positive group, compared with the control group. AMH level was significantly lower (3.57 ± 1.7 vs. 3.16 ± 3.34 ng/ml; *p* = 0.0002) and FSH level was significantly higher (7.05 ± 2.3 vs. 8.27 ± 4.09 mIU/ml; *p* = 0.04) in endocrine-positive group, compared with controls.

There were no significant differences in serum levels of LH, E2, prolactin, TSH, FT3, FT4, TT, DHEAS, androstendione, 17-OHP and SHBG between the endocrine negative and endocrine positive patients. Assessing carbohydrate metabolism, baseline glucose level (4.75 ± 0.47 vs. 5.05 ± 0.61 mmol/l; *p* = 0.003) at baseline insulin level (7.2 ± 2.38 vs. 10.13 ± 7.21 µU/ml; *p* = 0.003) and 120-min glucose level (5.08 ± 1.54 vs. 6.00 ± 1.59 mmol/l; *p* = 0.006) and 120-min insulin level (32.41 ± 13.7 vs. 69.4 ± 69.83 µU/ml; *p* = 0.01) were significantly higher in endocrine-positive group. No significant difference was observed in vitamin D levels between the two groups.

There was no significant difference between the two groups for the number of cycles performed.

Average number of retrieved oocytes, however, was significantly lower in endocrine-positive group, as compared with the control group (7.5 ± 3.92 vs. 5.42 ± 3.82; *p* = 0.0001).

Pregnancy rate (61.43% vs. 34.16%; *p* = 0.003) was also significantly lower in the endocrine-positive group, compared with the control group, and this difference (*p* = 0.0151) was observed in age-corrected rates, as well.

### Comparison of Patients with PCOS and Women Without Endocrinopathies

Average age was significantly lower in the PCOS group, as compared to the control group (32.32 ± 5.04 vs. 29.88 ± 4.61 years; *p* = 0.017). BMI value (22.88 ± 3.32 vs. 27.55 ± 5.93 kg/m^2^; *p* < 0.0001), serum triglyceride level (0.95 ± 0.48 vs. 1.34 ± 0.86, *p* = 0.03) and AMH level (3.5 ± 1.7 vs. 7.52 ± 4.18 ng/ml; *p* < 0.0001) were significantly higher in PCOS group as well. FSH level (7.05 ± 2.3 vs. 5.87 ± 1.43 mIU/l; *p* = 0.0061) was significantly lower and the LH level (6.35 ± 2.51 vs. 8.86 ± 5.38 mIU/ml; *p* = 0.0037) was significantly higher is PCOS group. However, a significant difference was not found in levels of E2, TSH, FT3, FT4, DHEAS and androstendione. Total testosterone (1.05 ± 0.45 vs. 1.81 ± 1.29 nmol/l; *p* = 0.0051) and 17-OHP (1.85 ± 1.08 vs. 3.18 ± 1.91 nmol/l; *p* = 0.0065) were significantly higher in PCOS group. Serum SHBG (67.23 ± 43.59 vs. 46.71 ± 27.74 nmol/l; *p* = 0.02) level was significantly lower in the endocrine-positive group.

Serum baseline glucose level (4.75 ± 0.47 vs. 5.18 ± 0.75 mmol/l; *p* = 0.0095) and baseline insulin level (7.2 ± 2.38 vs. 15.14 ± 11.82 µU/ml; *p* = 0.0001), 120-min glucose level (5.08 ± 1.54 vs. 6.19 ± 1.9 mmol/l; *p* = 0.01) and 120-min insulin level (32.41 ± 13.76 vs. 111.5 ± 99.28 µU/ml; *p* = 0.006) were significantly higher in study group, but there was no significant difference between glucose and insulin levels at 60 min. No significant difference was observed in vitamin D levels between the two groups.

There was no significant difference between the two groups for the number of cycles performed and the average number of retrieved oocytes in the PCOS group, compared with controls.

Pregnancy rate (61.43% vs. 40.0%; *p* = 0.061) was lower in the endocrine-positive group, compared with the controls (*p* = 0.001), but this difference was not significant. There was no significant difference (*p* = 0,061) in pregnancy rate. However, the difference in age correlated rate was significant (*p* = 0.0027) for those under 35 years old. In the subgroup of over 35, the age-correlated difference was not significant (*p* = 0.26).

### Comparison of Women with Hypothyroidism Without AITD and Women Without Endocrinopathies

The average age was significantly higher (32.32 ± 5.04 vs. 35.33 ± 5.15 years; *p* = 0.04), and AMH level was significantly lower (3.57 ± 1.7 vs. 2.87 ± 3.17 ng/ml; *p* = 0.02) in patients with hypothyroidism but without AITD, as compared to the control group. There was no significant difference in average BMI and in levels of FSH, LH, E2, prolactin, androgen levels, SHBG, TSH, free thyroid-hormones, vitamin-D, serum glucose and insulin between the two groups.

Furthermore, there was no significant difference between the two groups in the number of cycles performed. However, the number of retrieved oocytes was significantly lower in the hypothyroidism group (7.5 ± 3.92 vs. 5.0 ± 4.84; *p* = 0.0094).

The pregnancy rate was also lower in the hypothyroidism group (61.43% vs. 33.33%; *p* = 0.83), and this non-significant difference could also be observed in age-corrected rates as well (*p* = 0.13).

### Comparison of Women with Hashimoto’s Thyroiditis and Women Without Endocrinopathies

The average age was significantly higher (32.32 ± 5.04 vs. 36.13 ± 4.92 years; *p* = 0.0001) and AMH level was significantly lower in the AITD positive group (3.57 ± 1.7 vs. 2.81 ± 2.72 ng/ml; *p* = 0.009). There was no significant difference in BMI of the two groups. There were also no differences observed in start-of-cycle FSH, LH, E2, prolactin, androgen hormones, SHBG, vitamin D, TSH and FT3 levels. The FT4 level was higher within the normal range (1.13 ± 0.21 vs. 1.22 ± 0.22 ng/dl; *p* = 0.047) in the AITD-positive group.

Serum baseline glucose level (4.75 ± 0.47 vs. 5.2 ± 0.63 mmol/l; *p* = 0.0001) and baseline insulin level (7.2 ± 2.38 vs. 9.49 ± 5.46 µU/ml; *p* = 0.01) and 120-min glucose level (5.08 ± 1.54 vs. 5.93 ± 1.44 mmol/l; *p* = 0.01) and 120-min insulin level (32.41 ± 13.76 vs. 55.82 ± 38.08 µU/ml; *p* = 0.0025) were significantly higher in the study group, but there were no differences between glucose and insulin levels at 60 min.

There was no significant difference between the two groups for the number of cycles performed. However, the number of retrieved oocytes was significantly lower in the AITD group (7.5 ± 3.92 vs. 5.74 ± 3.72; *p* = 0.014). Pregnancy rate was significantly lower in the endocrine-positive group, compared to the control group (61.43% vs. 29.41%; *p* = 0.001), and this difference was significant in age-corrected rates as well (*p* = 0.0095).

### Comparison of Patients with DOR and Women Without Endocrinopathies

Average age (32.32 ± 5.04 vs. 38.16 ± 4.72 years; *p* < 0.0001) and BMI (22.88 ± 3.32 vs. 24.73 ± 4.34 kg/m^2^; *p* = 0.0125) was higher in DOR patients, while AMH level (3.57 ± 1.7 vs. 0.628 ± 0.35 ng/ml; *p* < 0.0001) was lower. The FSH level was higher (7.05 ± 2.3 vs. 11.29 ± 5.0 mIU/ml; *p* < 0.001) in the DOR group. No differences were found in levels of LH, E2, prolactin, TSH, FT3, FT4, androgen hormones and SHBG. However, serum baseline glucose level (4.75 ± 0.47 vs. 5.04 ± 0.52 mmol/l; *p* = 0.003) and 120-min glucose level (5.08 ± 1.54 vs. 5.99 ± 1.52 mmol/l; *p* = 0.01) was higher in the DOR group.

With no difference in the number of cycles performed, the average number of retrieved oocytes (7.5 ± 3.92 vs. 2.36 ± 1.45; *p* < 0.0001) was much less in the DOR group, compared to the endocrine negative patients. Pregnancy rate (61.43% vs. 21.82%; *p* < 0.001) was significantly lower in the DOR group, compared with controls, and this difference remained significant (*p* = 0.0109) in age-corrected rates as well.

### Comparison of Patients with Hyperprolactinaemia and Women Without Endocrinopathies

There were no differences in average age (32.32 ± 5.04 vs. 34.22 ± 5.5 years; *p* = 0.09) and BMI. AMH level was lower (3.57 ± 1.7 vs. 3.22 ± 3.14 ng/ml; *p* = 0.025), and prolactin level was slightly higher (14.17 ± 4.92 vs. 21.69 ± 14.3, *p* = 0.007) in patients with hyperprolactinaemia, both within the normal range. Further, no differences were observed in start-of-cycle FSH, LH, E2, TSH, FT3, FT4, androgen hormones, SHBG, vitamin D and serum glucose and insulin levels.

The number of cycles was significantly higher (1.61 ± 0.66 vs. 2.00 ± 0.81; *p* = 0.023) in the hyperprolactinaemia group without difference in the average number of oocytes retrieved. Pregnancy rate (61.43% vs. 29.03%; *p* = 0.005) was significantly lower, and this difference remained significant (*p* = 0.0072) in age-corrected cohort rates as well.

### Rare Endocrinological Disorders

Due to the low number of cases (Graves’ disease 3 cases, acute thyroiditis 1 case, thyroid cancer 1 case, hypogonadotropic hypogonadism 2 cases and congenital adrenal hyperplasia 1 case), no subgroup analysis of reproductive outcomes could be performed, after the first IVF cycle pregnancy was registered in 2 patients with Graves’ disease and in both cases with hypogonadotropic hypogonadism [22].

## Discussion

### Prevalence of Endocrinopathies and Their Effect on IVF Outcomes

In this prospective single-centre study, there was a high incidence of endocrine diseases among women undergoing IVF adversely impacting IVF success even after proper treatment. The average age (34 years), order of frequency and distribution of the primary indications for IVF for the 231 patients in this study were similar to the recent literature [23]: male factor (30%), DOR (34%), fallopian tube obstruction (18%), ovulatory dysfunction (14%), unknown (8%) AND endometriosis (6%). Thus, this patient population can be considered representative for further evaluation.

In our study, at least one endocrine disorder occurred in 69.7% (study group), which was the primary infertility factor in 37.5% of subjects, and a co-factor in 32.2%. Patients without endocrine disorders served as the control group. We found that the pregnancy rate after IVF treatment in the study group is reduced to almost half, as compared to the control group, despite optimal endocrine treatment before IVF. Our results will not explain a sole underlying mechanism, since the infertile patient population is complex with frequent coincidences of more than one disorder. However, through the subgroup analysis of our patients we try to highlight of the possible endocrine factors which might influence the IVF outcome negatively.

The relationship between aging and the decline of ovarian function is not a novel finding and may manifest.

through a number of plausible pathways other than endocrine abnormalities. The comparison of patient subgroups in terms of age was carried out with completeness of statistical description of patient data in mind, not as a way of supporting a hypothesis of a primary endocrine pathway. Since the negative impact of the higher age of the study group may play a role in this difference [24], we adjusted the calculation to age, but the difference remained significant.

Obesity, which also affects women of reproductive age, has been on the rise for the past 30 years [25]. Patients with endocrinological abnormalities have higher BMI and triglyceride level, mainly due to the simultaneous presence of PCOS and IR, as with significant differences in oral glucose tolerance test (OGTT) parameters, which cannot always be fully optimized despite intensive lifestyle therapy and pharmacological treatments.

There were also significant differences in ovarian reserve capacity (FSH, AMH and number of oocytes obtained) between the groups with and without endocrinological abnormalities, which is mainly attributable to patients with DOR. However, the differences in AMH and oocyte count were also strongly associated with thyroid autoimmunity [26].

Differences could not be observed in thyroid function between the two groups, since subclinical and clinical hypothyroidism as well as hyperthyroidism were corrected before the IVF treatment. The prevalence of thyroid disease in all patients in this study was 32.5%, which is higher than that in published literature [27], which could be due to our detailed procedure to test thyroid function.

No significant differences in androgenic hormones were detected between the groups, as hyperandrogenism had been optimized as much as possible pre-procedure, with higher testosterone levels — still within the normal range — in PCOS patients being the only exception.

Although the laboratory parameters had been normalized as much as it was possible, we registered a significantly lower pregnancy rate in the group of patients with endocrinopathies.

### Hypothyroidism and Thyroid Autoimmunity

Several authors attempted to find evidence to prove that hypothyroidism and thyroid autoimmunity have a negative impact on reproductive health. Hypothyroidism and the resulting hyperprolactinaemia cause infertility through the impaired pulsatile secretion of GnRH and its consequences, like anovulation. Thyroid hormone is also required for the proper synthesis of estrogen and progesterone. During stimulation treatment, high estrogen concentrations increase the quantity of thyroxine-binding globulin, which results in decreased fT4 levels and causes compensatory TSH elevation [28]. In light of this, the level of thyroid hormones may affect the success of IVF treatments. Appropriate thyroid hormone replacement is important prior to IVF treatment, with TSH level serving as the clinical indicator [29].

The mean age of patients with hypothyroidism without thyroid autoimmunity (AITD) was higher in the entire cohort, but there was no significant difference in BMI between both groups. However, AMH levels and the number of oocytes retrieved were significantly lower in the endocrine positive group, as compared to the control group, in which the patients’ age also played a role. Because hypothyroidism was appropriately corrected with thyroxine replacement, the values of thyroid function did not differ from those of the control group. The IVF results of patients having hypothyroidism without AITD did not differ from those in the control group, but the evaluation is limited by the relatively small number of cases in our sample.

Several authors have investigated the adverse impact of AITD on natural fertility and reproductive parameters. In a 2016 comprehensive literature review and meta-analysis, Busnelli et al. examined and defined the relation between AITD and IVF/ICSI cycle outcomes. The authors discussed the available evidence on AITD association with increasing age. The authors also showed that TSH levels were higher in AITD-positive patients in 6 of the 12 studies they reviewed, although no detailed survey of free thyroid hormone levels was performed. However, no significant differences were found regarding the number of oocytes retrieved, fertilization and implantation rates or the clinical pregnancy rate in AITD-positive patients. Nevertheless, the miscarriage rate was significantly higher in AITD-positive patients, compared with controls [30].

In our study, we found that the mean age of patients with AITD is significantly higher, with similar BMI values. With respect to the ovarian reserve, AMH and the number of oocytes retrieved were significantly lower in AITD group, without increase in FSH. No difference was found in the initial TSH value, as the aim was to keep the TSH value below 2.5 mIU/ml by thyroid hormone replacement. FT4 was found to be significantly higher in AITD-positive patients than in those without endocrinopathies, which could also be explained by thyroxine replacement. Despite maximal efforts to keep the TSH below the widely accepted threshold, a significantly lower clinical pregnancy rate was recorded in the AITD-positive group, compared to the control group, and the difference also remained after age correction.

#### PCOS

PCOS is considered not only to be an ovarian disease but also a multisystemic condition and, in addition to metabolic processes, it affects the hypothalamic-pituitary-ovarian axis, due to insulin resistance and hyperandrogenism. Beyond causing infertility, PCOS is characterized by obesity, metabolic dysfunctions and cardiovascular abnormalities and therefore requires complex treatment [31].

BMI and triglyceride level in the PCOS group were significantly higher, despite introducing lifestyle changes and medication, if necessary, to reduce IR, hypothyroidism and hyperprolactinaemia in these patients, for months and sometimes years before their IVF treatment. If regular ovulation could not be achieved even with the complex treatment supplemented with ovulation induction, the patient was diagnosed with chronic anovulation and enrolled in the IVF program. AMH produced by ovarian granulosa cells may be important in establishing the reproductive status of a patient with PCOS [32], although AMH values in patients with PCOS are typically high. In our study, AMH values were higher, but IVF treatments still resulted in a lower pregnancy rate, which can be attributed to poorer parameters of oocyte quality [33]. Higher LH and lower FSH levels have an adverse effect on the processes of oocyte maturation and fertilization, and a poorer embryo quality results in lower pregnancy rates and higher miscarriage rates.

The laboratory parameters of thyroid function did not show differences between patients with PCOS and the control group, as the TSH had also been adjusted to less than 2.5 mIU/l, in case hypothyroidism was associated with PCOS.

Despite the complex treatment introduced well before IVF, we have been unable to treat infertile patients with PCOS as effectively as those without endocrinopathies. Although the number of oocytes retrieved did not differ and the patients’ age was lower, the pregnancy rate was lower in patients younger than 35 years, even after age correction.

### Diminished Ovarian Reserve

Both terms DOR (diminished ovarian reserve) and POR (poor ovarian responder) refer to decreased ovarian reserve capacity, which is an increasingly common cause of infertility. The average age of patients with DOR requesting IVF treatment is increasing. As a result of environmental factors affecting women, the increasing incidence of DOR cannot be explained by ovarian aging alone. Ovarian surgery, smoking and chronic diseases, which become more common with advancing age, also play a role [34]. Additional factors such as genetic causes, metabolic and enzymatic abnormalities, toxic effects, infectious diseases and autoimmunity have already been identified as etiological factors in DOR. Diminished ovarian reserve capacity and poor responsiveness to gonadotropins also make IVF treatment difficult [35]. Several authors have confirmed that the pregnancy rates are lower in patients with DOR of different etiologies [36]. Recently, a consensus on the accurate identification and treatment of patients with diminished reserve capacity according to the Bologna criteria was established, using POSEIDON stratified groups. AMH and AFC values are of central importance in the diagnosis of DOR [37]. Having made the diagnosis, we examined the patients for polyglandular endocrinopathy. In addition to DOR, other endocrinological abnormalities were also present, most commonly Hashimoto’s thyroiditis, IR and hyperprolactinaemia. Although we did not find any difference in thyroid function values, we kept TSH below 2.5 mIU/l during the treatment. In patients with DOR, lipid profile was not different. The incidence of IR was close to 20%, which we tried to manage with lifestyle therapy, low-carbohydrate diet, exercise and, if necessary, using metformin. The higher age of our patients with DOR was also associated with higher FSH and lower AMH values. The number of oocytes retrieved and the pregnancy rate were significantly lower, and the difference remained even after correction for age, which potentially explains the negative effect of endocrinological abnormalities often associated with DOR.

### Hyperprolactinaemia

By inhibiting folliculogenesis at several targets, hyperprolactinaemia can lead to luteal-insufficiency and affects endometrial receptivity during IVF treatments, as well. High prolactin levels result in abnormal implantation and inadequate embryo development. Even transient hyperprolactinaemia has an adverse effect on the success of IVF treatment [38].

In nearly half of our patients with hyperprolactinaemia, the etiology was unclear (functional hyperprolactinaemia); the rest had PCOS, drugs, and pituitary microadenoma as underlying causes. There was no difference between patients with hyperprolactinaemia, and healthy patients as far as their age, BMI, reproductive hormones, OGTT results, or thyroid functions were considered. AMH levels were slightly lower, and, even after correction using bromocriptine, the prolactin levels remained higher than in the control group, which may explain the lower pregnancy rate in the test group despite both values being within the normal range in both groups.

In regard of the limitations of this study, consecutive patient enrolment may influence the data integrity and may cause selection and treatment bias. However, a single-centre based study using systematic endocrine evaluation, uniform endocrine and IVF treatment may reflect the real-life conditions and strengthen the validity of the data reported. Since pregnancy outcomes were not included in this study, longer term effects of treated endocrine diseases on IVF pregnancies are not reported here.

## Conclusion

In this paper, we point out that endocrine diseases are often present in patients participating in IVF programs with a higher prevalence at elevated age. ART outcome is adversely affected by various underlying concomitant endocrinopathies regardless of optimal treatment and despite proper correction of laboratory parameters.

## Data Availability

Data and materials are available.
